# Structured Fruit Cube Snack of BRS Vitoria Grape with Gala Apple: Phenolic Composition and Sensory Attributes

**DOI:** 10.3390/molecules29215205

**Published:** 2024-11-04

**Authors:** Yara Paula Nishiyama-Hortense, Carolina Olivati, Victoria Diniz Shimizu-Marin, Ana Carolina Gonçales, Natália Soares Janzantti, Roberto Da Silva, Ellen Silva Lago-Vanzela, Sergio Gómez-Alonso

**Affiliations:** 1 Institute of Biosciences, Humanities and Exact Sciences (Ibilce), Campus São José do Rio Preto, São Paulo State University (UNESP), Rua Cristóvão Colombo n° 2265, São José do Rio Preto 15054-000, Brazil; carolinaolivati@gmail.com (C.O.); victoriadshimizu@gmail.com (V.D.S.-M.); ana.goncales@unesp.br (A.C.G.); natalia.soares-janzantti@unesp.br (N.S.J.); dasilva@ibilce.unesp.br (R.D.S.); ellen.sl.vanzela@unesp.br (E.S.L.-V.); 2 Faculty of Chemical Sciences and Technologies, University of Castilla-La Mancha (UCLM), Avenue Camilo José Cela s/n, 13071 Ciudad Real, Spain; sergio.gomez@uclm.es; 3 Regional Institute for Applied Scientific Research (IRICA), University of Castilla-La Mancha (UCLM), Avenue Camilo José Cela s/n, 13071 Ciudad Real, Spain

**Keywords:** Brazilian hybrid grape, HPLC-DAD-ESI-MS^n^, natural snacks, rate-all-that-apply, anthocyanins, flavonols, hydroxycinnamic acid derivatives

## Abstract

This study developed a structured fruit cube (FC) snack using only natural ingredients, specifically red grape and apple, without hydrocolloids and sucrose. After development, physicochemical characterization and analysis of phenolic compounds (PCs), including anthocyanins, flavonols, and hydroxycinnamic acid derivatives, using HPLC-DAD-ESI-MS/MS, were conducted. Sensory quality was also assessed through an acceptance and sensory profile analysis using the rate-all-that-apply methodology. The results showed that the FC had physicochemical characteristics similar to other structured fruits that use hydrocolloids. Additionally, they presented a complex composition of PCs, predominantly including anthocyanins derived from malvidin (tri-substituted methoxylated anthocyanins), notably cumarylated ones. Flavonols compounds comprised the 3-glucoside series of myricetin, quercetin, laricitrin, kaempferol, isorhamnetin, and syringetin; the 3-galactoside series of myricetin and quercetin; and the 3-glucuronic acid series of myricetin and quercitin, along with rutin. The presence of caftaric acid, coutaric acid, fertaric acid, and *p*-coumaroyl-glucose was also detected, alongside caffeic acid-*O*-glucoside 1, caffeic acid-*O*-glucoside 2, chlorogenic acid, 4-O-*p*-coumaroylquinic acid, and dicaffeoylquinic acid. In conclusion, the selection of natural ingredients was technologically suitable for obtaining an FC. Despite using conventional drying at 60 °C, the product showed notable concentrations of PCs and also achieved great sensory acceptance.

## 1. Introduction

Considering the increasing concern of the population in adopting healthier diets, there is a rising demand for convenient, ready-to-eat dehydrated products that incorporate fruit-based ingredients into their formulation, offering an alternative for integrating these foods into the diet. These products are often rich in diverse nutrients, possess functional properties, and are lower in calories compared to other snacks [1,2,3,4,5]. Among these, fruit cube snacks (FC) stand out as a promising option, not only as a convenient fruit-based snack but also as an attractive and versatile ingredient for the food industry.

FC can incorporate a variety of fruits [6], and selecting ingredients that enhance the product’s appeal and add health benefits is crucial. In this context, red grapes have been extensively studied as ingredients due to their attractive color, pleasant flavor, and nutritional enhancement through phenolic compounds (PCs) [7,8,9,10,11]. PCs are bioactive compounds known for their potential health benefits, especially related to their antioxidant properties and promotion of cardiovascular health [12]. The seedless Brazilian grape BRS Vitoria has an interesting variety and quantity of PCs, including a diversity of anthocyanins, such as monoglucosides, diglucosides, and acylated forms; flavonols; hydroxycinnamic acid derivatives (HCAD); flavan-3-ols; proanthocyanidins; and stilbenes [13,14].

In the development of this type of product, the structure is generally achieved by using fruit juice or pulps combined with hydrocolloids such as pectin, agar, and gelatin or edible gums such as gellan, konjac, xanthan, carrageenan, and guar [6,15,16,17] and may contain sugars and sweeteners [4,18]. In this research, the authors propose using apples as a natural texturizer due to their significant pectin content. Chandel et al. [19] report that the pectin content in apple pomace is generally about 10–15% on a dry matter basis, and this content depends on the variety, origin, and harvest of the fruit [20]. Previous studies have evaluated the use of apples as a base for producing similar products, such as fruit bars [21,22]. Vázquez-Sánchez et al. [4] successfully produced apple leather enriched with Acáchul (*Ardisia compressa* Kunth) powder without the use of any hydrocolloids.

As far as is known, there are no previous studies on the phenolic compositions of FC produced exclusively from a mixture of fruits (grape and apple) with their peels without the addition of sucrose and hydrocolloids. Furthermore, the authors propose a simple, accessible, and reproducible processing method. This could provide a viable option for processing whole fruit, especially those that do not meet the quality requirements for fresh market sale or are surplus production during the harvest season. Moreover, this aligns with the principles of the circular economy and the short marketing chains [23,24,25].

To assess the nutritional and functional value of the FC, a basic physicochemical characterization, as well as the determination of the qualitative and quantitative composition of the main PCs (anthocyanins, flavonols, and HCAD) through high-performance liquid chromatography with a diode array detector coupled with mass spectrometry (HPLC-DAD) with electrospray ionization and ion trap analyzer (ESI-MS^n^), were conducted. Lastly, to evaluate the sensory quality of the product, the analysis of acceptance and sensory profile of the FC using the rate-all-that-apply (RATA) methodology was employed.

## 2. Results

### 2.1. Physicochemical Characteristics of FC

In the present study, the FC produced in duplicate (FC1 and FC2) showed the physicochemical characteristics presented in Table 1.

### 2.2. HPLC-DAD-ESI-MS/MS Identification and Quantification of PCs of FC

Table 2 displays the anthocyanins identified in the FC produced in duplicate (FC1 and FC2), their molar, and their total concentration expressed in mg malvidin-3-glc·kg^−1^ and mg malvidin-3-5-glc·kg^−1^, along with the usual information pertaining to MS and MS/MS spectra (*m*/*z*) for these compounds. In the FC for both processes, 33 anthocyanin derivatives were identified from the 5 anthocyanidins (delphinidin, cyanidin, petunidin, peonidin, and malvidin, in monoglycosylated and diglycosylated forms). For monoglycosylated derivatives, all five main anthocyanidins were found in non-acylated, acetylated, and cumarylated forms. Additionally, their *p*-coumaroyl derivatives (in *cis*-conformations) of delphinidin, petunidin, and malvidin were also observed.

Caffeylated forms were only found for cyanidin and malvidin. Regarding diglycosylated derivatives, the following were identified: non-acylated derivatives of cyanidin, petunidin, peonidin, and malvidin; *p*-coumaroyl derivatives of peonidin and malvidin, as well as delphinidin and petunidin (in *cis*- and *trans*-conformations); acetyl derivatives of peonidin and malvidin; and caffeyl derivates of malvidin.

The total anthocyanin concentration in the FC, expressed in mg·kg^−1^ as malvidin-3,5-diglc equivalents observed, was 2984.53 (FC1) and 3168.23 (FC2), with no significant difference between batches. Concerning the flavonol profile (Table 3) described for FC1 and FC2, a total of 13 compounds were detected. These monoglycosylated flavonols are derivatives of the six main possible flavonol aglycones: myricetin, quercetin, laricitrin, kaempferol, isorhamnetin, and syringetin. The quercetin type (average of 74.92% for FC1 and 78.24% for FC2) was found in the highest proportions, followed by the myricetin type (average of 12.18% for FC1 and 11.66% for FC2), laricitrin type (average of 3.44% for FC1 and 3.50% for FC2), kaempferol type (average of 5.57% for FC1 and 1.98% for FC2), isorhamnetin type (average of 1.90% for FC1 and 2.57% for FC2), and syringetin type (average of 1.99% for FC1 and 2.05% for FC2).

The total flavonol concentration recorded for FC expressed in mg·kg^−1^ as quercetin-3-glc equivalents was 442.10 (FC1) and 485.04 (FC2), with no significant difference observed between productions.

In the FC samples, nine HCADs were detected (Table 4). The majority proportions, in molar ratio, of HCAD were for dicaffeoylquinic acid (23.70% for FC1 and 16.44% for FC2) and fertaric acid (21.85% for FC1 and 25.54% for FC2). The total HCAD concentration recorded for FC, expressed in mg·kg^−1^ as caftaric acid equivalent, was 612.80% for FC1 and 674.97% for FC2, with no significant difference observed between productions.

### 2.3. Sensory Analysis

**Figure 1:**
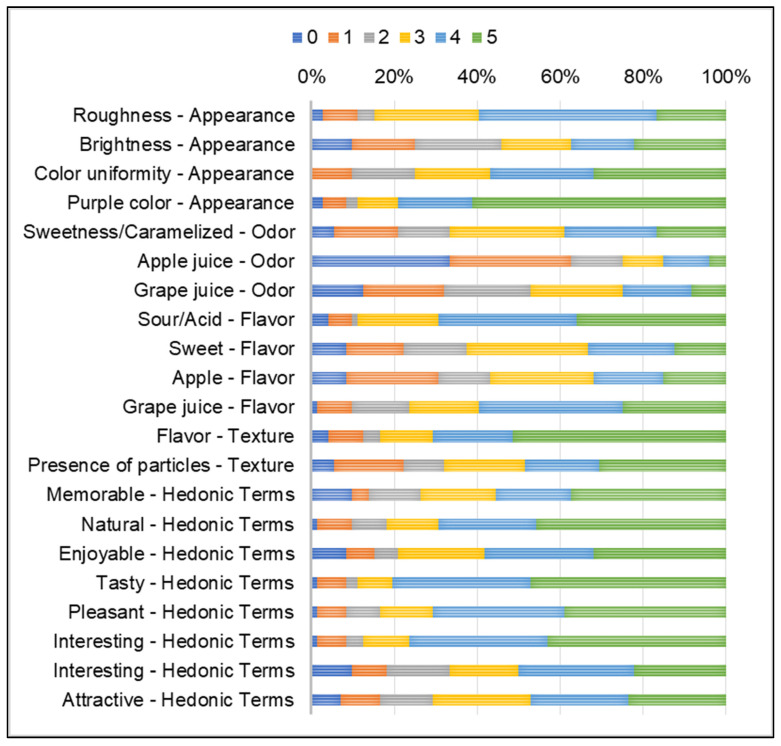
Proportions of the scores obtained for each evaluated term (scale: 0—not applicable; 1—hardly applicable; 2 to 4 — intermediate values without classification; 5—very applicable).

The RATA technique, coupled with acceptance testing, enables the characterization of a novel product using untrained consumers and an evaluation of its acceptance. Table 5 displays the average scores of sensory descriptors and acceptance for the FC, while Figure 1 shows the proportions of each score on the scale for each evaluated term. Through the RATA analysis, the FC was primarily described (on a scale ranging from 3 to 5) by terms such as *roughness* (3.47), *color uniformity* (3.54), and *purple color* (4.18) regarding appearance; *sour/acidic* (3.81) and *grape juice/grape* (3.50) regarding flavor; *soft* (3.89), and the *presence of particles* (3.19) regarding texture; along with all hedonic terms like *memorable* (3.43), *natural* (3.86), *enjoyable* (3.46), *tasty* (4.07), *pleasant* (3.83), *interesting* (3.97), *sophisticated* (3.11), and *attractive* (3.18).

**Figure 2:**
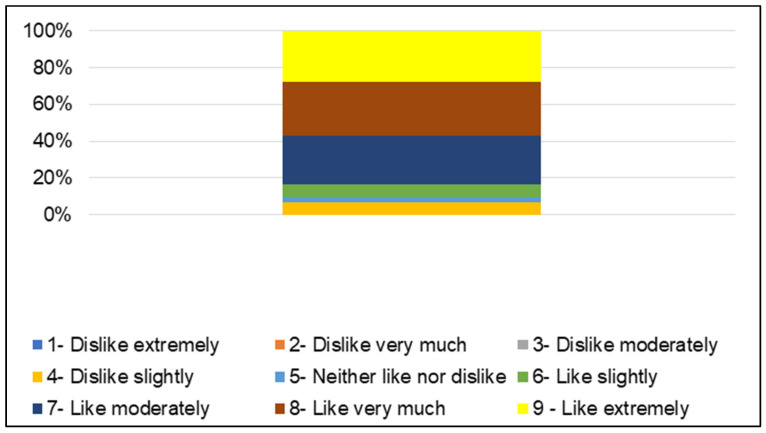
Mean score (*n* = 72) of the global acceptance of the fruit cube snack (FC).

The majority of consumers (56.94%) stated that they liked very much or extremely liked (hedonic values between 8 and 9) the FC (Figure 2), which is clearly reflected in the mean global acceptance score of the product (7.51 ± 1.40) (Table 5). All consumers rated the FC positively, with no one expressing dislike.

**Figure 3:**
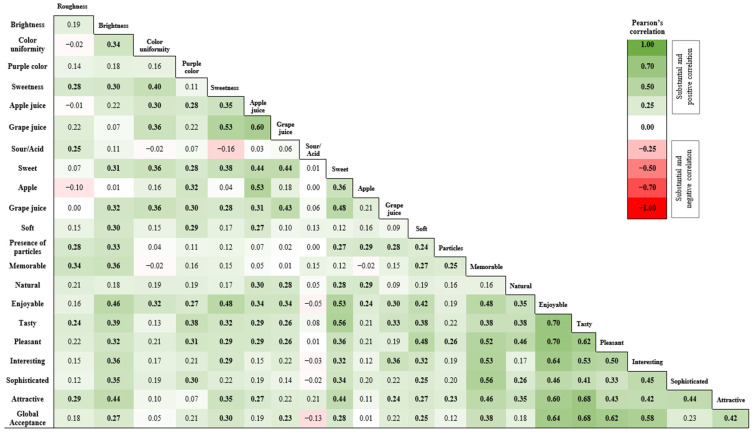
Heatmap of Pearson’s correlations considering RATA Sensory Descriptors and Global Acceptance of Sensory Analysis.

A Pearson Correlation Analysis with a 95% confidence level (*p* ≤ 0.05) and a substantial correlation of r > 0.5 was conducted to determine which sensory descriptors were correlated with the overall acceptance of the FC and to examine the correlations between the terms used. The results are shown in Figure 3

The *global acceptance* of the FC had a substantial correlation (r > 0.5) with the hedonic terms *interesting* (0.58), *enjoyable* (0.64), *tasty* (0.68), and *pleasant* (0.62). Among the descriptor terms, a substantial correlation was evident between the odor term *sweetness/caramelized* and the odor term *grape juice* (0.53); between the odor term *apple juice,* the odor term *grape juice* (0.60), and the flavor term *apple* (0.53); between the flavor term *sweet* and the hedonic terms *enjoyable* (0.53) and *tasty* (0.56); between the hedonic term *memorable* and the hedonic terms *pleasant* (0.52), *interesting* (0.53), and *sophisticated* (0.56); between the hedonic term *enjoyable* and the hedonic terms tasty (0.70), *pleasant* (0.70), *interesting* (0.64), and *attractive* (0.60); and between the hedonic term *tasty* and the hedonic terms *pleasant* (0.62), *interesting* (0.53), and *attractive* (0.68).

## 3. Discussion

The FC obtained from the two different processes (FC1 and FC2) did not exhibit significant differences in their physicochemical characteristics (Table 1), indicating good process reproducibility with regard to these important parameters related to their quality.

There is limited information about structured fruit products made without added hydrocolloids and sugars. However, the FC developed in this study achieved the characteristics expected for this type of product. The developed FC exhibited Wa and moisture within the range reported by other authors by similar products structured with different hydrocolloids (values between 0.7 and 0.9% and 20 and 30%, respectively) [26,27]. In Brazil, the specific requirements for Dried or Dehydrated Fruit Products (excluding tender dried fruits) indicate a maximum moisture content of 25% (g·100 g^−1^) [28]. Research indicates that moisture and, mainly Wa, plays a crucial role in extending the shelf life and preserving microbiological stability during the storage of dehydrated products [4,29,30], the findings of the present study regarding FC suggest that they may be deemed sufficiently stable to withstand commercialization without microbiological deterioration. It is worth noting that international regulations regarding the standards of identity and quality for dehydrated fruit-derived products vary depending on the country and region. However, there are some widely recognized guidelines adopted by many countries and international organizations, such as the Food and Drug Administration (FDA), Codex Alimentarius, and the Regulations of the European Union.

To aid in microbiological stability and product structuring due to pectin gelatinization in apples, the formulations were supplemented with tartaric acid, reflected in the acidity and pH values of the final products (Table 1). Vázquez-Sanchez et al. [4] reported that the low Wa (about 0.45) and pH values, which remained stable during the storage period studied (12 weeks at 25 °C), were the primary factors responsible for maintaining the microbiological stability of apple leathers enriched with acáchul. Depending on the ingredients comprising the formulation and due to interactions occurring primarily between the vegetable matrix and the hydrocolloids during processing, it is observed in the literature that there are products with varying pH values (ranging between 3.2 and 4.6) [26,27,31]. 

Regarding the qualitative and quantitative determination of PCs identified in FC snacks, in Table 2, significant differences (*p* ≤ 0.05) were observed among the anthocyanins in the products obtained through different processes (FC1 and FC2). However, it is evident that anthocyanins derived from malvidin (trisubstituted methoxylated anthocyanins), particularly cumarylated ones, were predominant in both. These anthocyanins found in the products are also predominant in the grape used in the formulation [14]. Studies on grape processing indicate the greater stability to thermal degradation of malvidin compared to other aglycones like cyanidin and delphinidin. Among these anthocyanins, the *p*-coumaroyl group stands out, as it contributes to the formation of both inter- and intra-molecular co-pigmentation complexes, thereby enhancing the stability of the molecules [32,33,34].

There were no significant differences in the molar percentages of flavonols identified in FC obtained through different processes (FC1 and FC2). Based on flavonol 3-O-glycosides previously reported in the same grape variety used in the preparation of these FC snacks, the detected monoglycosylated flavonol derivatives include 3-glucoside (3-glc) series of myricetin, quercetin, laricitrin, kaempferol, isorhamnetin, and syringetin; the 3-galactoside (3-gal) series of myricetin and quercetin; and the 3-glucuronic acid (3-glcU) series of myricetin and quercetin. The only diglycoside flavonol identified in the present study is known as rutin (quercetin-3-(6′′-rhamnosyl)-glucoside; quercetin-3-rutin), a derivative from quercetin aglycone, which has also been previously reported in the BRS Vitoria grape [14].

Other derivatives of the quercetin aglycone, specifically 3-xyloside (3-xyl) and 3-rhamnoside (3-rhm) of the quercetin aglycone, respectively, named reinutrin and quercitrin, were also identified. These compounds have been previously reported in Gala apples produced in Brazil [35,36]; therefore, it is presumed that these flavonols in FC snacks originated from this fruit, as they are not typically found in grapes.

Similarly to flavonols, the HCAD profile showed no significant variation among the studied FC. Of the identified HCAD compounds (Table 4), caftaric acid, coutaric acid, fertaric acid, and *p*-coumaroyl-glucose were also found in BRS Vitoria grapes [14], and 4-*O*-*p*-coumaroylquinic acid and chlorogenic acid were found in Gala apples [35,36]. In fact, these last two compounds may be the dominant phenolic acids reported in apples [37]. Although there are currently no records for Gala apples, both caffeic acid hexoside isomers and dicaffeoylquinic acid have been identified in other apple varieties. Caffeic acid hexoside isomers were determined by Horvacki et al. [38] in about 24 different apple cultivars produced in Serbia and by Arraibi et al. [39] in Spanish and Belgian apple pomace. Dicaffeoylquinic acid was identified by He et al. [40] in juices and ciders from six different apple cultivars grown in Finland. Therefore, it is plausible that apples are the source of these compounds in the FC.

The results demonstrate that despite conventional drying at 60 °C, the produced item presented a noteworthy concentration of PCs. The qualitative composition of anthocyanins, flavonols, and HCAD highlights that the developed snacks have diverse compounds with functional properties, demonstrating their role in promoting human health and well-being.

Studies indicate that some of these compounds can act as antioxidants capable of scavenging free radicals from cells and participating in the regeneration of vitamin E and ascorbic acid. Additionally, antioxidant activity can be mediated by multiple mechanisms, such as the elimination or reduction of reactive species; chelation of metal ions, which are capable of catalyzing lipid peroxidation; and inhibition of enzymes involved in oxidative stress. PCs are also implicated in safeguarding cellular constituents against oxidative damage, thereby mitigating the risk of chronic diseases such as cardiovascular ailments, cancer, and diabetes [12,41]. Moreover, numerous studies have underscored the anti-inflammatory effects of several identified compounds, aiding in attenuating systemic inflammation associated with various health conditions, including autoimmune disorders, obesity, and cardiovascular diseases. Additionally, these compounds exhibit specific beneficial effects, such as enhancing gut health and neuroprotective properties that contribute to maintaining brain health and reducing the risk of neurodegenerative diseases [42,43,44,45,46,47,48,49]. Therefore, the diversity of PCs present in FCs is crucial for providing a wide range of health benefits, underscoring the importance of including these products in a balanced diet.

With the physicochemical results demonstrating good process reproducibility, the FCs obtained from both processes (FC1 and FC2) were combined for the sensory analysis. In the sensory analysis, RATA results indicated most consumers described the FC snacks as having a rough appearance, uniform color, purple color, sour/acid and grape juice, and soft texture with the presence of particles. The majority of consumers also described the FC snacks as memorable, natural, enjoyable, tasty, pleasant, interesting, sophisticated, and attractive, all hedonic terms. The RATA technique also showed that most consumers did not consider the descriptor terms related to odor (“sweetness/caramelized”, “apple juice”, and “grape juice”) appropriate to describe the sample (scale ranging lower than 3), probably because it has a slightly or very characteristic odor, which is not related to the terms suggested in the study.

In analyzing Pearson’s linear correlation (Figure 3), it can be seen that although all the hedonic terms were considered applicable to describe the samples, the global acceptance just had a substantial (r > 0.5) and significant (*p* ≤ 0.05) correlation with the terms enjoyable, tasty, pleasant and interesting. It is noteworthy that these terms also correlated with each other, demonstrating that for consumers, there is a positive relationship between them; in other words, if a product is perceived as tastier, it is likely to be more pleasurable for them as well.

Given the importance of these terms to product acceptance, it is interesting to note that the terms pleasant and tasty also correlated positively with sweet flavor. The determination obtained by the RATA analysis that the sweet flavor was considered inadequate to describe the samples indicates a necessity to incorporate additional ingredients to enhance the sweetness of the product, which could be achieved with some natural products such as honey. It is also important to acknowledge some considerations for future research. Although the sensory analysis provided valuable insights, the sample size (*n* = 72) may limit the generalizability of these findings, particularly in relation to the correlations made. Therefore, conducting a larger sensory evaluation would be beneficial for further confirming consumer preferences. Additionally, it is necessary to evaluate the shelf life and storage conditions to ensure that the snacks maintain their quality, nutritional properties, and sensory appeal over time, thereby supporting their potential for commercialization.

The findings of this study have significant implications for the food industry. The developed methodology demonstrates strong potential for scalability, enabling the production of healthier snack alternatives that can be tailored to various markets. By utilizing natural ingredients and eliminating hydrocolloids, this approach represents an innovative strategy for crafting FC snacks that meet the increasing consumer demand for nutritious and convenient food products. Consequently, this research not only enhances the understanding of the physicochemical and sensory characteristics of the snacks but also underscores their importance in fostering healthier dietary habits.

## 4. Material and Methods

### 4.1. Chemicals

During the experiments, analytical-grade chemicals (>99%) and ultrapure water (Milli-Q system, Merk-Millipore, Darmstadt, Germany) were used. Phenolic compound analysis was conducted using LC-MS grade solvents obtained from Fisher Scientific (Madrid, Spain), specifically acetonitrile, formic acid, and methanol. The chemical standards malvidin-3-glucoside (malvidin-3-glc), malvidin-3,5-diglucoside (malvidin-3,5-glc), and *trans*-caftaric acid were obtained from Phytolab (Vestenbergsgreuth, Germany). Caffeic acid was from Sigma-Aldrich (Tres Cantos, Madrid, Spain). Kaempferol, quercetin, isorhamnetin, myricetin, syringetin, and 3-glucoside (3-glc) of kaempferol, quercetin, isorhamnetin, and syringetin and the 3-galactosides (3-gal) of myricetin, kaempferol, quercetin, and isorhamnetin were obtained from Extrasynthese (Genay, France). The commercially unavailable standards myricetin-3-glc, quercetin-3-glucuronic acid (quercetin-3-glcU), and laricitrin-3-glc had previously been isolated from Petit Verdot grape skins [50]. 

### 4.2. Grape and Apple

Grapes and apples were used as the main ingredients in the making of the FC. The ripe BRS Vitoria seedless grapes (4 kg) were harvested in the city of Marialva, state of Paraná (South Brazil), located at 23°29′ South and 51°47′ West, and 570 m above sea level (refer to WGS84 datum [51] (World Geodetic System, 1984) and donated by the Experimental Station of Tropical Viticulture (Embrapa, Jales, Brazil). A batch of Gala apples (*Malus domestica*) (7 Kg) was purchased at the local market in São José do Rio Preto, São Paulo state, Brazil. The physicochemical characteristics from representative samples of the batches of grapes and apples were determined in triplicate, according to the AOAC [52]. For the grapes, the following results were obtained: moisture, 78.63 ± 0.36%; hydrogen potential (pH), 3.62 ± 0.02; titratable acidity (TA), 0.72 ± 0.02 g of tartaric acid per 100 g of grape; and soluble solids (SS), 21.50 ± 0.05 °Brix at 25 °C. For the apples, the physicochemical characteristics were as follows: moisture, 84.32 ± 0.21%; pH, 3.88 ± 0.13; TA, 0.28 ± 0.01 g of malic acid per 100 g of apple; and SS, 16.20 ± 2.60 °Brix at 25 °C. The results are averages ± standard deviations from a minimum of three independent determinations.

### 4.3. FC Preparation

**Figure 4:**
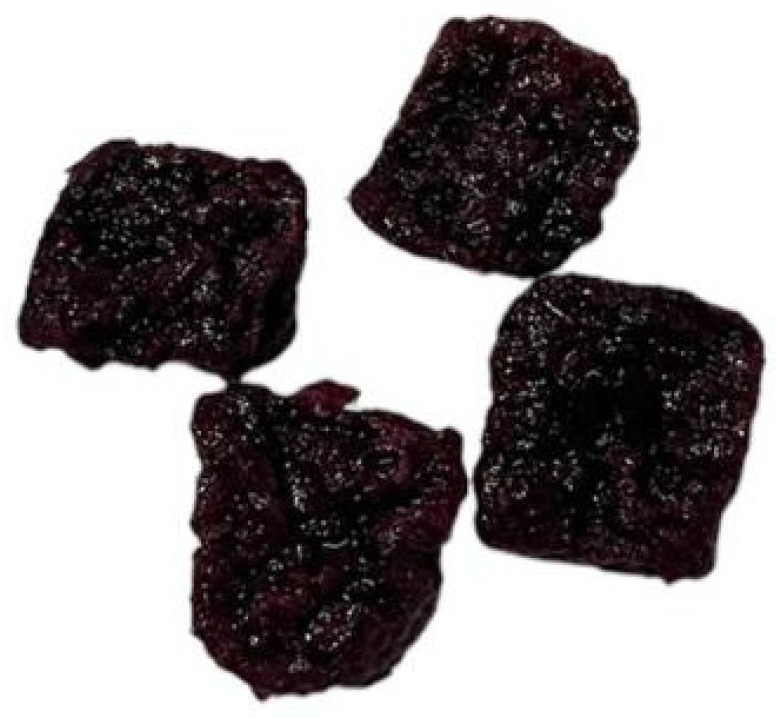
Developed fruit cube (FC) snacks.

The apples and grapes were previously sanitized with chlorinated water. The apples were cut into cubes with peels, subjected to blanching by steam for 5 min, and then immediately cooled to stop further cooking. The formulation of the FC consisted of a mixture of blanched apple cubes (60%, *w*/*w*) and grape berries (40%, *w*/*w*) in their whole form. This mixture was homogenized using a Philips Walita blender, with tartaric acid added to adjust the pH of 3.3, placed in silicone trays, and dehydrated in a convective drier with hot air (60 °C; 1 m·s^−1^). The dehydration was concluded after a reduction of at least 75% weight, controlled by weighing the trays containing the formulation. The FCs (1.0 cm side) (Figure 4) were then put into polyethylene bags and kept in frozen storage until analysis. The FC was produced in duplicate, resulting in 2 products (FC1 and FC2).

### 4.4. Evaluation of Physicochemical Characteristics of FC

The physical–chemical characteristics (moisture, pH, TA, and water activity (Wa)) of the FC1 and FC2 were determined in triplicate (*n* = 3), according to the AOAC [51]. Moisture was determined gravimetrically with an oven (315 SE, Fanem^®^, Guarulhos, Brazil) at 105 °C; the result was expressed as g of water per 100 g of wet sample (%). For pH and TA analysis, a pH meter (TEC-5, Tecnal, Piracicaba, Brazil) was used. The TA result was expressed as tartaric acid g·100 g^−1^. The Aw, at 25 °C, was determined using an electric hygrometer (Axair Ltd., Novasina, Aw Sprint, Lachen, Switzerland).

### 4.5. Evaluation of HPLC-DAD-ESI-MS/MS Identification and Quantification of PCs of FC

Detailed determination of PCs (anthocyanins, flavonols, and HCAD) was conducted using previously described methods [53]. First, samples of 15.0 g (*n* = 3) from FC1 and FC2 were crushed under freezing conditions in a redesigned batch mill (A 10 basic, IKA, Königswinter, Germany) along with solid carbon dioxide (CO_2_) until reaching a powdered state. Subsequently, 10.0 g of these powdered samples (*n* = 3) was homogenized (Heidolph DIAX 900, Merck, Rahway, NJ, USA) with 50 mL of a solvent mixture of methanol, water, and formic acid (70:28.5:1.5, *v*/*v*). Four repeat extractions were used for recovery of the PCs, similar to the methodology described by Rebello et al. [53]. All of the supernatants recovered from the four extractions were combined and dried in a rotary evaporator (35 °C), and each volume was made up to 100 mL with sodium chloride (NaCl) (0.1N). All extractions were realized in triplicate.

For anthocyanin analysis, aliquots (5 mL) from extracts of the FC were submitted to an extraction using SPE-C18 cartridges (Waters, Milford, CT, USA) to remove the sugars and other polar compounds, as described by Olivati et al. [54], and injected (10 µL) into the chromatographic column. Prior to the analysis of flavonols and HCAD analysis, to obtain an anthocyanin-free and sugars-free fraction, aliquots (3 mL) from prepared samples of FC were extracted in Bond Elut Plexa PCX cartridges (Agilent Technologies, Santa Clara, CA, USA) [13] and injected (20 μL) into the chromatographic column.

The HPLC separation, identification, and quantification of anthocyanins, flavonols, and HCAD were carried out using the same conditions described by Rebello et al. [53] on an Agilent 1100 Series system (Agilent Technologies, Waldbronn, Germany), equipped with DAD (G1315B) and an LC/MSD Trap VL (G2445C VL) ESI-MS^n^ system and coupled with an Agilent Chem Station (version B.01.03) data-processing station. The mass spectra data were processed with Agilent LC/MS Trap software (version 5.3). For quantification, DAD-chromatograms were extracted at 520 (anthocyanins), 360 (flavonols), and 320 nm (HCAD).

Anthocyanins, flavonols, and HCAD identified in the samples were presented quantitatively in molar ratio (%) and normalized to the total content. The sum of all compounds of the same type was quantitatively reported as the total concentration, with results expressed, respectively, as malvidin-3-glc for anthocyanin 3-glucosides and malvidin-3,5-diglc for anthocyanin 3,5-diglucosides; mg equivalents of quercetin-3-glc for the flavonol 3-glucosides; and caftaric acid for the HCAD.

All the standards were used for identification and quantitation through calibration curves covering the expected concentration ranges. So, using a previous method to analyze anthocyanins, flavonols, and HCAD of Brazilian grapes by direct injection in HPLC-DAD [53], a reversed-phase column Zorbax Eclipse XDB-C18 (2.1 × 150 mm; 3.5 μm particle; Agilent Technologies, Waldbronn, Germany) was also used to obtain the curves of the standards (malvidin-3-glc, malvidim-3,5-diglic, quercetin-3-glic, and caftaric acid) with the respective R^2^, LOD, and LOQ were reported in the Supplementary Material (Table S1), as well as the analysis time and ionization mode for each phenolic class analyzed.

### 4.6. Evaluation of Sensory Analysis

For the sensory analysis, the FCs obtained from both processes (FC1 and FC2) were combined into a single sample batch. Seventy-two consumers aged between 18 and 61 years old, 94% no smokers and 68% female, who liked and consumed products containing grapes and/or apples, evaluated the FC snacks through an acceptance test and descriptive analysis using the RATA method [55]. In the acceptance test [56], the three-digit number-encoded FC snacks were evaluated by consumers using a structured nine-point hedonic scale (scale: 1—extremely dislike, 2—dislike very much, 3—dislike moderately, 4—dislike slightly, 5—neither like nor dislike, 6—like slightly, 7—like moderately, 8—like very much, 9— extremely like). In the RATA test, a descriptive team first developed the sensory descriptors (4 of these for appearance, 3 for odor, 4 for taste, 2 for texture, and 8 for hedonic terms). Then, consumers were instructed to indicate the intensity (scale: 0—not applicable; 1—little applicable; 5—very applicable) of the FC snacks, as shown in the form provided in the Supplementary Material (Figure S1). The descriptors presented were randomized [57]. Consumers performed sensory analyses in individual booths lit by incandescent light. The study was approved by the local research ethics committee under the Certificate of Presentation for Ethical Consideration (CAAE) nº 70679517.3.0000.5466.

### 4.7. Data Analysis

To compare the phenolic composition between the produced FCs (FC1 and FC2), Student’s *t*-test was used at a significance level of 0.05 (α = 0.05). Pearson’s linear correlation analysis was performed on the results of sensory analysis, considering a significance level of 0.05 (*p* ≤ 0.05) and a substantial correlation r ≥ 0.5. All analyses were performed using IBM SPSS Statistics V 20.0 software (SPSS Inc., IBM, Armonk, NY, USA).

## 5. Conclusions

The use of natural ingredients in the formulation proved to be technologically suitable for FC production, resulting in a product with good structure, attractive coloration, and excellent physicochemical characteristics considered optimal for the dehydrated fruit-derived products category. Despite employing conventional drying at 60 °C, the resulting FC showed significant concentrations of PCs. Whereby the anthocyanins showed a higher concentration of diglycosylated compounds, mainly derived from malvidin, the flavonols showed a higher concentration of compounds derived from quercitin, and the HCADs showed the highest concentrations of dicaffeoylquinic acid and fertaric acid. Sensory evaluation using RATA techniques highlighted that consumers described the FC snacks with most of the proposed sensory descriptors (for appearance, flavor, texture, and hedonic terms). Although the correlation of pleasant and tasty with sweet flavor suggests that improvements in this area could enhance consumer satisfaction, this is a product with great potential as a healthy snack due to its concentration of PCs and its high global acceptance.

## Figures and Tables

**Table 1:** Chemical and physical characteristics of fruit cube snack (FC), given as mean values ± standard deviation (*n* = 3).

Determinations	FC1	FC2
Moisture (%)	25.23 ± 1.50a	24.86 ± 0.58a
Wa	0.76 ± 0.00a	0.75 ± 0.00a
TA (acid g·100 g^−1^)	0.02 ± 0.00a	0.02 ± 0.00a
pH	3.29 ± 0.00a	3.32 ± 0.00a

FC1 and FC2 represent two distinct productions of the fruit cube snack. TA, total acidity; pH, hydrogen potential; Wa, water activity. a—different lowercase letters on the same line indicate significant differences between the samples according to Student’s *t*-test (*p* ≤ 0.05).

**Table 2:** Anthocyanins in fruit cube snack (FC) according to HPLC-DAD-ESI-MS/MS (positive ionization mode), molar profiles, and total concentration given as mean values ± standard deviation (*n* = 3).

Assignation ^1^	Molecular Ion; Product Ions (*m*/*z*)	Molar Ratio (%)	
		FC1	FC2
Delphinidin-3-glc	465; 303	2.01 ± 0.48b	5.22 ± 0.38a
Cyanidin-3-glc	449; 287	2.10 ± 0.31b	3.89 ± 0.13a
Petunidin-3-glc	479; 317	0.90 ± 0.76b	6.21 ± 0.37a
Peonidin-3-glc	463; 301	3.59 ± 0.44b	5.12 ± 0.01a
Malvidin-3-glc	493; 331	10.07 ± 1.12a	11.91 ± 0.09a
Delphinidin-3-acglc	507; 303	0.20 ± 0.02b	0.41 ± 0.02a
Cyanidin-3-acglc	491; 287	0.61 ± 0.14a	0.74 ± 0.10a
Petunidin-3-acglc	521; 317	0.22 ± 0.03b	0.41 ± 0.02a
Peonidin-3-acglc	505; 301	0.19 ± 0.06a	0.15 ± 0.01a
Malvidin-3-acglc	535; 331	1.33 ± 0.07a	1.46 ± 0.02a
Cyanidin-3-cfglc	611; 287	1.43 ± 0.16a	1.55 ± 0.08a
Malvidin-3-cfglc	655; 331	1.68 ± 0.12a	1.69 ± 0.10a
*c*-delphinidin-3-cmglc	611; 303	0.08 ± 0.02b	0.15 ± 0.01a
*c*-petunidin-3-cmglc	625; 317	0.49 ± 0.07a	0.58 ± 0.04a
*c*-malvidin-3-cmglc	639; 331	0.72 ± 0.05a	0.68 ± 0.06a
*t*-delphinidin-3-cmglc	611; 303	2.56 ± 0.31b	4.48 ± 0.20a
Cyanidin-3-cmglc	595; 287	2.36 ± 0.42b	5.39 ± 0.35a
*t*-petunidin-3-cmglc	625; 317	3.75 ± 0.29b	5.25 ± 0.22a
Peonidin-3-cmglc	609; 301	2.07 ± 0.13b	2.80 ± 0.08a
*t*-malvidin-3-cmglc	639; 331	9.32 ± 0.41a	9.30 ± 0.27a
cyanidin-3,5-diglc	611; 449, 287	0.46 ± 0.01b	0.57 ± 0.01a
Petunidin-3,5-diglc	641; 479, 317	0.98 ± 0.06b	1.39 ± 0.02a
Peonidin-3,5-diglc	641; 479, 317	7.24 ± 0.38a	6.27 ± 0.19b
Malvidin-3,5-diglc	655; 493, 331	21.75 ± 0.09a	13.10 ± 0.72b
Peonidin-3-acglc-5glc	667; 505, 301	0.21 ± 0.02a	0.14 ± 0.01b
Malvidin-3-acglc-5glc	697; 535, 493, 331	1.24 ± 0.15a	0.60 ± 0.02b
Malvidin-3-cfglc-5-glc	817; 655, 331	2.85 ± 0.33a	1.11 ± 0.10b
*c*-delphinidin-3-cmglc-5-glc	773; 611, 465, 303	0.68 ± 0.13a	0.76 ± 0.19a
*c*-petunidin-3-cmglc-5-glc	787; 625, 479, 317	0.06 ± 0.03a	0.04 ± 0.04a
*t*-delphinidin-3-cmglc-5-glc	773; 611, 465, 303	3.14 ± 0.29a	2.37 ± 0.09b
*t*-petunidin-3-cmglc-5-glc	787; 625, 479, 317	1.70 ± 0.53a	0.50 ± 0.05a
peonidin-3-cmglc-5-glc	771; 609, 463, 301	0.26 ± 0.05a	0.33 ± 0.03a
malvidin-3-cmglc-5-glc	801; 639, 493, 331	13.74 ± 0.89a	5.46 ± 0.15a
Total (mg malvidin-3-glc·kg^−1^)		2000.62 ± 156.48a	2123.76 ± 63.12a
Total (mg malvidin-3,5-glc·kg^−1^)		2984.53 ± 233.43a	3168.23 ± 94.17a

FC1 and FC2 represent two distinct productions of the fruit cube snack. ^1^ Assignation: 3,5-diglc, 3,5-diglucosides; 3-acglc-5-glc, 3-(6″-acetyl)-glucoside-5-glucoside; 3-cmglc-5-glc, 3-(6″-*p*-coumaroyl)-glucoside-5-glucoside; 3-cfglc-5-glc, 3-(6″-*p*-caffeoyl)-glucoside-5-glucoside; 3-glc, 3-glucoside; 3-acglc, 3-(6″-acetyl)-glucoside; 3-cmglc, 3-(6″-*p*-coumaroyl)-glucoside; 3-cfglc, 3-(6″-*p*- caffeoyl)-glucoside; pent, pentosideo; *c-*, cis isomer; *t-*; trans isomer; Total expression in mg of malvidin-3-glucoside or mg of malvidin-3,5-diglucoside for kg of FC snack. a, b—different lowercase letters on the same line indicate significant differences between the samples according to Student’s *t*-test (*p* ≤ 0.05).

**Table 3:** Flavonols in fruit cube snack (FC) according to HPLC-DAD-ESI-MS/MS (negative ionization mode), molar profiles (percentage of each individual flavonol regarding the total content), and total concentration, given as mean values ± standard deviation (*n* = 3).

Assignation ^1^	Molecular Ion; Product Ions (*m*/*z*)	Molar Ratio (%)	
		FC1	FC2
Myricetin-3-glcU	493; 317	1.69 ± 0.65a	0.77 ± 0.20a
Myricetin-3-gal	479; 317	4.84 ± 0.63a	8.44 ± 0.02a
Myricetin-3-glc	479; 317	5.65 ± 2.84a	2.44 ± 0.13a
Quercetin-3-gal	463; 301	26.96 ± 2.27a	20.262 ± 2.90a
Quercetin-3-glcU	477; 301	10.86 ± 3.54a	15.56 ± 2.66a
Quercetin-3-glc	463; 301	19.57 ± 0.78a	21.03 ± 0.51a
Quercetin-3-rut	609; 301	4.29 ± 0.16a	2.38 ± 0.19a
Quercetin-3-xyl	433; 301	3.66 ± 0.47a	6.86 ± 1.49a
Quercetin-3-rhm	447; 301	9.58 ± 0.40a	12.15 ± 2.77a
Laricitrin-3-glc	493; 331	3.44 ± 0.62a	3.50 ± 0.37a
Kaempferol-3-glc	447; 285	5.57 ± 3.10a	1.98 ± 0.43a
Isorhamnetin-3-glc	477; 315	1.90 ± 0.16a	2.57 ± 0.54a
Syringetin-3-glc	507; 345	1.99 ± 0.07a	2.05 ± 0.31a
Total (% of flavonol)		100	100
Quercetin type		74.92 ± 6.81a	78.24 ± 2.00a
Myricetin type		12.18 ± 2.86a	11.66 ± 0.35a
Laricitrin type		3.44 ± 0.62a	3.50 ± 0.37a
Kaempferol type		5.57 ± 3.10a	1.98 ± 0.43a
Isorhamnetin type		1.90 ± 0.16a	2.57 ± 0.54a
Syringetin type		1.99 ± 0.07a	2.05 ± 0.31a
Total (% by type of flavonol)		100	100
Total (mg of quercetin-3-glc·kg^−1^)		442.10 ± 11.55a	485.04 ± 81.76a

FC1 and FC2 represent two distinct productions of the fruit cube snack. ^1^ Assignation: 3-glcU, 3-glucuronide acid; 3-gal, 3-galactoside; 3-glc, 3-glucoside; quercetin-3-rut, quercetin-3-(6′′-rhamnosyl)-glucoside (quercetin-3-rutine); 3-xyl, 3-xyloside; 3-rhm, 3-rhamnose (in molar percentage). Total expression in mg of quercetin-3-glc (quercitin-3-glucoside) for kg of FC snack. a—different lowercase letters on the same line indicate significant differences between the samples according to Student’s *t*-test (*p* ≤ 0.05).

**Table 4:** Hydroxycinnamic acid derivatives (HCAD) in fruit cube snack (FC) according to HPLC-DAD-ESI-MS/MS (negative ionization mode), molar profiles (percentage of each HCAD regarding the total content), and total concentration, given as mean values ± standard deviation (*n* = 3).

Assignation	Molecular Ion; Product Ions (*m*/*z*)	Molar Ratio (%)	
		FC1	FC2
Caftaric acid	311; 179, 149, 135	2.42 ± 0.16a	2.67 ± 0.68a
Caffeic acid-*O*-glucoside 1	341; 179	7.67 ± 2.45a	10.43 ± 1.10a
Caffeic acid-*O*-glucoside 2	341; 179	3.50 ± 0.60a	4.06 ± 0.85a
Dicaffeoylquinic acid	515; 353, 191	23.70 ± 2.04a	16.44 ± 4.28a
Chlorogenic acid	353, 191	15.78 ± 2.51a	14.86 ± 2.10a
Coutaric acid	295; 163, 149, 119	1.86 ± 0.68a	1.92 ± 0.45a
Fertaric acid	325; 193, 149	21.85 ± 2.09a	25.54 ± 2.99a
*p*-Coumaroyl-glucose	325; 163, 145	12.88 ± 1.12a	15.89 ± 2.98a
4-*O*-*p*-Coumaroylquinic acid	339; 337, 173	10.33 ± 1.01a	8.20 ± 0.95a
Total (mg caftaric acid·kg^−1^)		674.97 ± 116.95a	612.80 ± 103.95a

FC1 and FC2 represent two distinct productions of the fruit cube snack. Total expressed in mg of caftaric acid for kg of FC snack. a—different lowercase letters on the same line indicate significant differences between the samples according to Student’s *t*-test (*p* ≤ 0.05).

**Table 5:** Means (±standard deviation, *n* = 72) attributed to sensory descriptors, hedonic terms, and acceptance of the fruit cube snack (FC).

Sensory Analysis		
Descriptors Terms ^1^		Mean ± Standard Deviation
Appearance	Roughness	3.47 ± 1.23
	Brightness	2.79 ± 1.65
	Color uniformity	3.54 ± 1.34
	Purple color	4.18 ± 1.33
Odor	Sweetness/Caramelized	2.96 ± 1.47
	Apple juice	1.49 ± 1.52
	Grape juice	2.36 ± 1.49
Flavor	Sour/Acid	3.81 ± 1.33
	Sweet	2.78 ± 1.47
	Apple	2.65 ± 1.57
	Grape juice	3.50 ± 1.31
Texture	Soft	3.89 ± 1.50
	Presence of particles	3.19 ± 1.63
Hedonic Terms	Memorable	3.43 ± 1.64
	Natural	3.86 ± 1.38
	Enjoyable	3.46 ± 1.56
	Tasty	4.07 ± 1.24
	Pleasant	3.83 ± 1.30
	Interesting	3.97 ± 1.26
	Sophisticated	3.11 ± 1.60
	Attractive	3.18 ± 1.52
Global Acceptance ^2^		7.51 ± 1.40

^1^ Scale: 0—not applicable; 1—hardly applicable; 5—very applicable; ^2^ Scale: 1—extremely dislike, 2—dislike very much, 3—dislike moderately, 4—dislike slightly, 5—neither like nor dislike, 6—like slightly, 7—like moderately, 8—like very much, 9—extremely like.

## Data Availability

The data presented in this study are available upon request from the corresponding author.

## Supplementary Materials

The following supporting information can be downloaded at: https://www.mdpi.com/article/10.3390/molecules29215205/s1, Table S1. Calibration curves, R^2^, LOD, LOQ, analysis time, and ionization mode for each phenolic class analyzed. Figure S1. Form with descriptive terms used for evaluation of the structured fruit cube (FC).

## Abbreviations

3,5-glc	3,5-diglucoside.
3-acglc	3-(6″-acetyl)-glucoside.
3-acglc-5-glc	3-(6″-acetyl)-glucoside-5-glucoside.
3-cfglc	3-(6″-*p*- caffeoyl)-glucoside.
3-cfglc-5-glc	3-(6″-p-caffeoyl)-glucoside-5-glucoside.
3-cmglc	3-(6″-*p*-coumaroyl)-glucoside.
3-cmglc-5-glc	3-(6″-*p*-coumaroyl)-glucoside-5-glucoside.
3-gal	3-galactoside.
3-glc	3-glucoside.
3-glcU	3-glucuronic acid.
3-rhm	3-rhamnose.
3-xyl	3-xyloside.
c	*cis* isomer.
CO_2_	Carbon dioxide.
ESI-MS^n^	Electrospray ionization and ion trap analyzer.
FC	Fruit cube snack.
gal	3-galactoside.
HCAD	Hydroxycinnamic acid derivatives.
HPLC-DAD	Liquid chromatography with diode array detector coupled with mass spectrometry.
*m*/*z*	MS/MS spectra.
NaCl	Sodium chloride.
ND	Not detected.
PCs	Phenolic compounds.
pent	Pentosideo.
pH	Hydrogen potential.
Quercetin-3-rut	Quercetin-3-(6′′-rhamnosyl)-glucoside or rutin.
RATA	Rate-all-that-apply.
SS	Soluble solids.
t	*trans* isomer.
TA	Titratable acidity.
Wa	Water activity.
