# Supplementary material for: Structured Fruit Cube Snack of BRS Vitoria Grape with Gala Apple: Phenolic Composition and Sensory Attributes
Source: Molecules. 2024 Nov 4;29(21):5205. doi: 10.3390/molecules29215205 (PMC11547337; doi:10.3390/molecules29215205)
Supplement: Supplementary file 1 [file molecules-29-05205-s001.zip › Supplementary material _Figure S1.pdf]

Figure S1. Form with descriptive terms used for evaluation of the structured fruit cube (FC).

Name: ____ Data:_______ **Sample:________**

Evaluate the STRUCTURED FRUIT CUBE (dehydrated product containing only grapes and apples without the addition of additives) and indicate with an X how, in your opinion, the terms do or do not apply to the sample.

	**0 Not applicable**	**1 Little applicable**	**2**	**3**	**4**	**5 Very applicable**
**Appearance**						
Roughness	( )	( )	( )	( )	( )	( )
Color uniformity	( )	( )	( )	( )	( )	( )
Brightness	( )	( )	( )	( )	( )	( )
Purple color	( )	( )	( )	( )	( )	( )
**Odor**						
Sweetness/Caramelized	( )	( )	( )	( )	( )	( )
Apple juice	( )	( )	( )	( )	( )	( )
Grape juice	( )	( )	( )	( )	( )	( )
**Flavor**						
Sour/Acid	( )	( )	( )	( )	( )	( )
Sweet	( )	( )	( )	( )	( )	( )
Apple	( )	( )	( )	( )	( )	( )
Grape juice	( )	( )	( )	( )	( )	( )
**Texture**						
Soft	( )	( )	( )	( )	( )	( )
Presence of particles	( )	( )	( )	( )	( )	( )
**Hedonic Terms**						
Memorable	( )	( )	( )	( )	( )	( )
Natural	( )	( )	( )	( )	( )	( )
Enjoyable	( )	( )	( )	( )	( )	( )
Tasty	( )	( )	( )	( )	( )	( )
Pleasant	( )	( )	( )	( )	( )	( )
Interesting	( )	( )	( )	( )	( )	( )
Sophisticated	( )	( )	( )	( )	( )	( )
Attractive	( )	( )	( )	( )	( )	( )

Comments: ____
