# Supplementary material for: Structured Fruit Cube Snack of BRS Vitoria Grape with Gala Apple: Phenolic Composition and Sensory Attributes
Source: Molecules. 2024 Nov 4;29(21):5205. doi: 10.3390/molecules29215205 (PMC11547337; doi:10.3390/molecules29215205)
Supplement: Supplementary file 1 [file molecules-29-05205-s001.zip › Supplementary material _Table S1.pdf]

Table S1. Calibration curves, R^2, LOD, LOQ, analysis time, and ionization mode for each phenolic class analyzed.

Compound	Calibration curve[1,2]	R^2	LOD (ppm)	LOQ (ppm)	Analysis time (min)[2]	Ionization mode[2]
malvidin-3-glucoside	y = 0.0058x + 0.7146	0.9998	0.014	0.047	45.000	Positive
malvidin-3,5-diglucoside	y = 0.0091x + 0.7146	0.9997	0.029	0.097	45.000	Positive
quercetin-3-glucoside	y = 0.0053x + 0.0009	0.9998	0.027	0.089	64.000	Negative
caftaric acid	y = 0.0033x - 0.2715	0.9996	0.037	0.124	64.000	Negative

[1]Y= area units; x=ppm; [2]A reversed-phase column Zorbax Eclipse XDB-C18 (2.1 × 150 mm; 3.5 µm particle; Agilent, Germany), thermostated at 40 °C, with flow rate of 0.19 mL/min was used.
